# Supplementary figures and images for: The Utility of Data Collected as Part of Australia’s Aboriginal and Torres Strait Islander Health Performance Framework
Source: Int J Environ Res Public Health. 2024 Mar 13;21(3):340. doi: 10.3390/ijerph21030340 (PMC10970423; doi:10.3390/ijerph21030340)

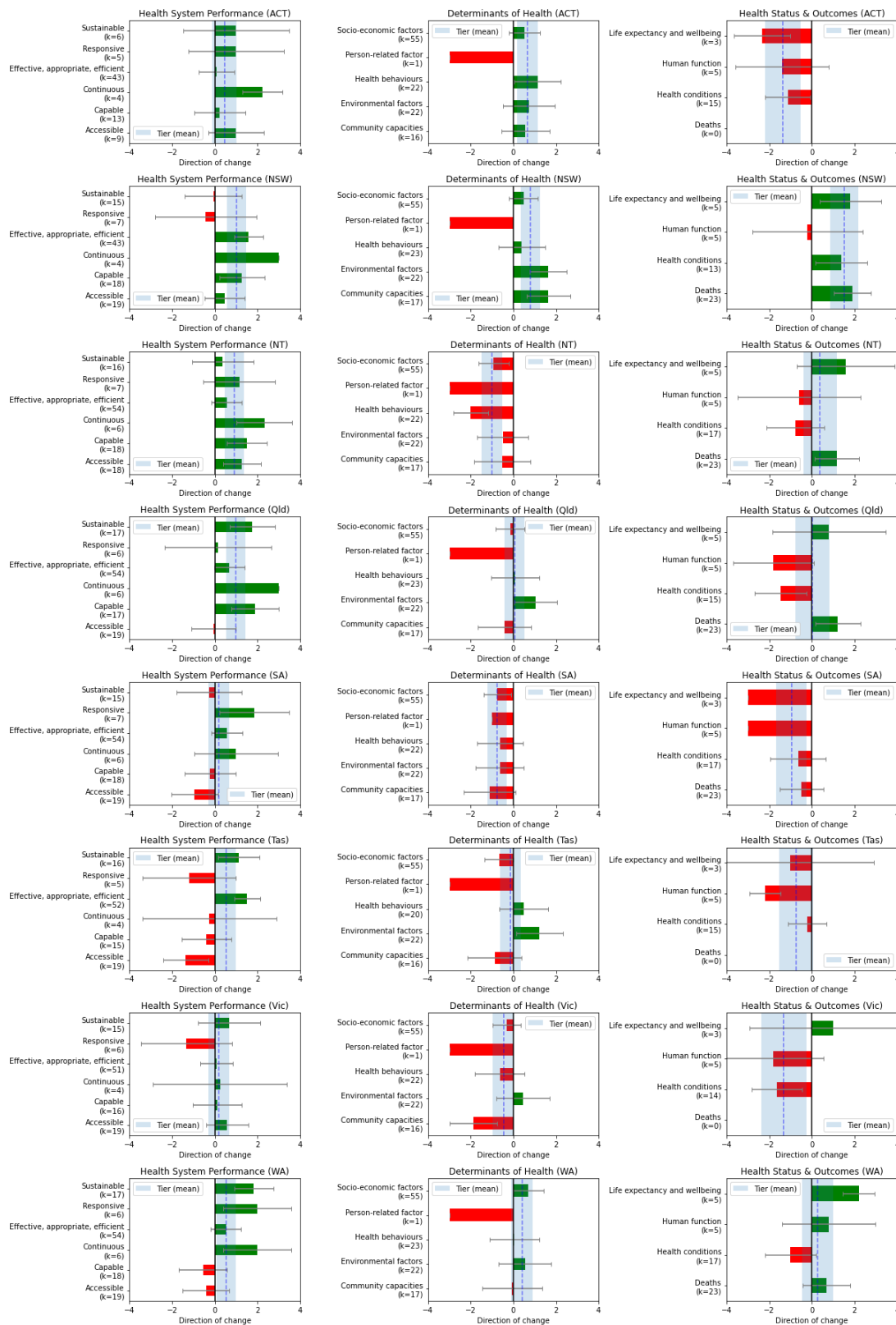

Figure S1. Example dashboard for monitoring improvements between 2017 and 2020 HPF reports.

Supplement: Supplementary file 1 [file ijerph-21-00340-s001.zip › ijerph-2845279-supplementary.pdf]
